# Supplementary material for: Effects of RDL GABA Receptor Point Mutants on Susceptibility to Meta-Diamide and Isoxazoline Insecticides in Drosophila melanogaster
Source: Insects. 2024 May 6;15(5):334. doi: 10.3390/insects15050334 (PMC11122182; doi:10.3390/insects15050334)
Supplement: Supplementary file 1 [file insects-15-00334-s001.zip › insects-2975174-supplementary.pdf]

Supplementary Material

Effects of RDL GABA receptor point mutants on susceptibility to meta-diamide and isoxazoline insecticides in *Drosophila melanogaster*

Authors:

Tianhao Zhou[1], Weiping Wu[1], Suhan Ma[1], Jie Chen[3], Jia Huang[1] and Xiaomu Qiao[1,2*]

Affiliations:

[1]Ministry of Agriculture Key Laboratory of Molecular Biology of Crop Pathogens and Insects, Institute of Insect Sciences, Zhejiang University, Hangzhou 310058, China

[2]Xianghu Laboratory, Hangzhou 311231, China

[3]Collaborative Innovation Center of Green Pesticide, National Joint Engineering Laboratory of Biopesticide Preparation, Zhejiang A&F University State Key Laboratory of Subtropical Silviculture, School of Forestry and Biotechnology, Zhejiang A & F University, Hangzhou 311300, China

***Correspondence:** xiaomu_qiao@163.com

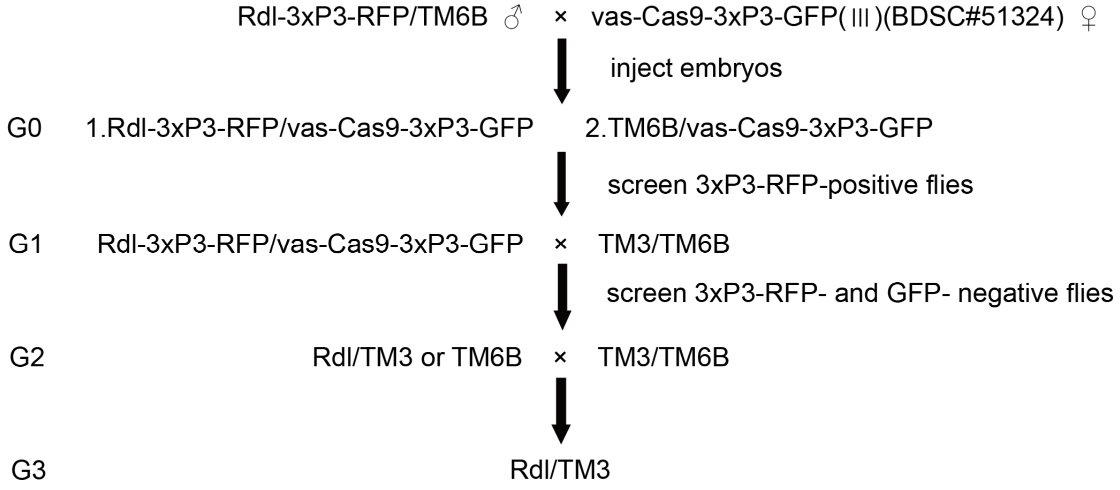

Supplementary Figure S1. Schematic of the strategy for generating mutant alleles of *Rdl* using a two-step CRISPR–Cas9 gene editing method. The crossing methods employed to generate knock-in lines are depicted.

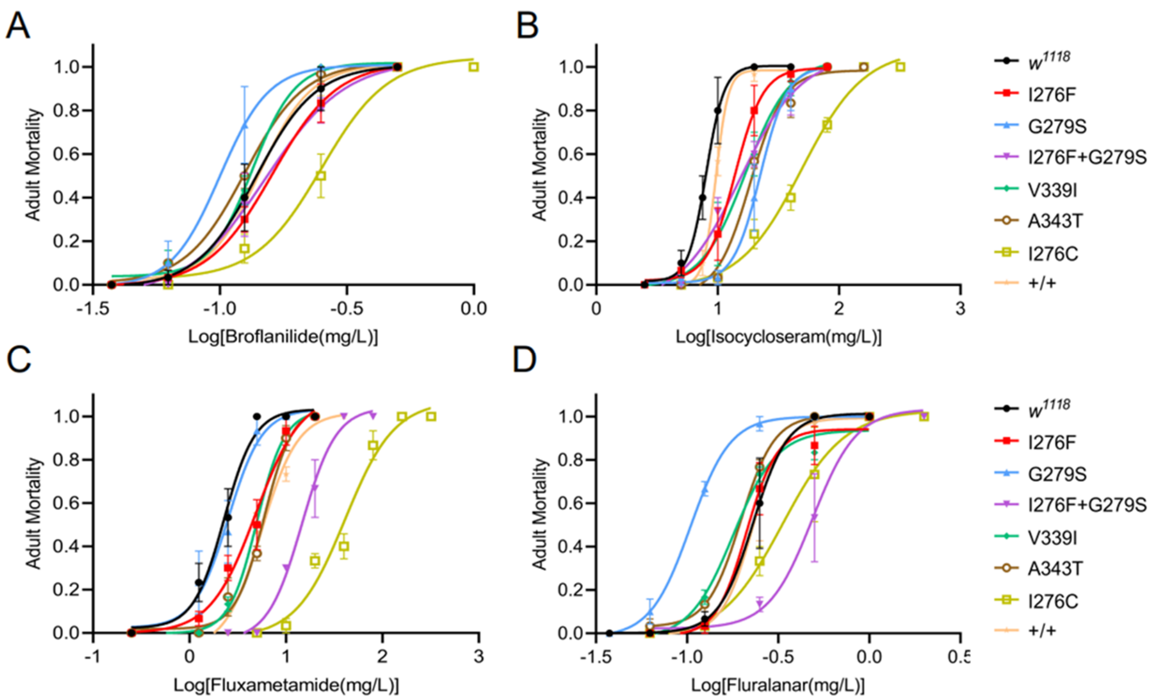

Supplementary Figure S2. Nonlinear log-dose mortality data for (A) broflanilide, (B) isocycloseram, (C) fluxametamide and (D) fluralaner against six *Drosophila Rdl* homozygous mutants and two controls. Mortality (0–1 means 0–100% in terms of percentage) of control and mutant female adults after 48 hours of exposure to increasing concentrations of insecticides. Error bars represent standard deviations. n = 3-4 trials, 3 replicates per trial.

Supplementary Table S1. ***Drosophila melanogaster*** **mutant lines generated in this study.**

Stock name	Donor plasmid	Parental strain
w^{1118}; *Rdl*^{I276C}	pUC57-Rdl-I276C	*w*^{1118}; *Rdl*^{3xP3RFP} male × *w*^{1118}; *vas-Cas9* (#51324) female
w^{1118}; *Rdl*^{G335M}	pUC57-Rdl-G335M	*w*^{1118}; *Rdl*^{3xP3RFP} male × *w*^{1118}; *vas-Cas9* (#51324) female

Supplementary Table S2. RDL TM1 and TM3 region (exon 7) sequences of wild-type and knock-in *Drosophila melanogaster* lines (engineered point mutations are highlighted in red background)

Line	Sequence
A (w^{1118}: wild-type)	GCAACTATTCGCGTTTAGCCTGCGAAATTCAGTTCGTGCGTTCGATGGGCTACTACCTTATA<br>CAAATCTACATACCCTCTGGACTGATCGTTATTATATCATGGGTATCATTTTGGCTCAATCGC<br>AATGCAACGCCGGCGCGTGTGGCGCTCGGTGTGACAACCGTGTTGACAATGACCACTTT<br>GATGTCGTCAACAAATGCAGCGCTGCCAAAGATTTCGTACGTCAAATCGATTGACGTCTAT<br>CTGGGAACATGCTTCGTTATGGTCTTTGCCAGTCTACTGG
I276C<br>(w^{1118}; Rdl^{I276C})	GCAACTATTCGCGTTTAGCCTGCGAAATTCAGTTCGTGCGTTCGATGGGCTACTACCTTATA<br>CAAATCTACTGTCCCTCTGGACTGATCGTTATTATATCATGGGTATCATTTTGGCTCAATCGC<br>AATGCAACGCCGGCGCGTGTGGCGCTCGGTGTGACAACCGTGTTGACAATGACCACTTT<br>GATGTCGTCAACAAATGCAGCGCTGCCAAAGATTTCGTACGTCAAATCGATTGACGTCTAT<br>CTGGGAACATGCTTCGTTATGGTCTTTGCCAGTCTACTGG
G335M<br>(w^{1118}; Rdl^{G335M})	GCAACTATTCGCGTTTAGCCTGCGAAATTCAGTTCGTGCGTTCGATGGGCTACTACCTTATA<br>CAAATCTACATACCCTCTGGACTGATCGTTATTATATCATGGGTATCATTTTGGCTCAATCGC<br>AATGCAACGCCGGCGCGTGTGGCGCTCGGTGTGACAACCGTGTTGACAATGACCACTTT<br>GATGTCGTCAACAAATGCAGCGCTGCCAAAGATTTCGTACGTCAAATCGATTGACGTCTAT<br>CTGATGACATGCTTCGTTATGGTCTTTGCCAGTCTACTGG

Supplementary Table S3. gRNAs used in the study.

gRNA name	gRNA sequences (5'-3')
gRNA-1	GCGACGCTTCGTTATCAGGC
gRNA-2	AACTAACAATGTTTCCAGGA
gRNA-3	GTCTTGTAGAGTCTTCGGGG
gRNA-4	GATGAAGGTTGATAGAACAG

Supplementary Table S4. Primers used in the study.

Primer name	Primer sequences (5’-3’)
DmRdlTM-F	GTGCGTTCGATGGGCTACTA
DmRdlTM-R	CGCTCAATGGATTGTCGGTG
